# The impact of hospital language on the rate of in-hospital harm. A retrospective cohort study of home care recipients in Ontario, Canada

**DOI:** 10.1186/s12913-020-05213-6

**Published:** 2020-04-21

**Authors:** Michael Reaume, Ricardo Batista, Robert Talarico, Emily Rhodes, Eva Guerin, Sarah Carson, Denis Prud’homme, Peter Tanuseputro

**Affiliations:** 1 Faculty of Medicine, University of Ottawa, 451 Smyth Rd, Ottawa, ON K1H 8L1 Canada; 2 Institut du Savoir Montfort, Ottawa, Canada; 3 Department of Medicine, Ottawa Hospital Research Institute, Ottawa, Canada; 4 ICES, Ottawa, Canada; 5 Faculty of Health Sciences, University of Ottawa, Ottawa, Canada; 6 Bruyère Research Institute, Ottawa, Canada

**Keywords:** Harmful events, Language barriers, Language concordance, Language discordance, Linguistic minorities, Patient safety

## Abstract

**Background:**

Patients who live in minority language situations are generally more likely to experience poor health outcomes, including harmful events. The delivery of healthcare services in a language-concordant environment has been shown to mitigate the risk of poor health outcomes related to chronic disease management in primary care. However, data assessing the impact of language-concordance on the risk of in-hospital harm are lacking. We conducted a population-based study to determine whether admission to a language-discordant hospital is a risk factor for in-hospital harm.

**Methods:**

We used linked administrative health records to establish a retrospective cohort of home care recipients (from 2007 to 2015) who were admitted to a hospital in Eastern or North-Eastern Ontario, Canada. Patient language (obtained from home care assessments) was coded as English (Anglophone group), French (Francophone group), or other (Allophone group); hospital language (English or bilingual) was obtained using language designation status according to the *French Language Services Act*. We identified in-hospital harmful events using the Hospital Harm Indicator developed by the Canadian Institute for Health Information.

**Results:**

The proportion of hospitalizations with at least 1 harmful event was greater for Allophones (7.63%) than for Anglophones (6.29%, *p* <  0.001) and Francophones (6.15%, *p* <  0.001). Overall, Allophones admitted to hospitals required by law to provide services in both French and English (*bilingual hospitals*) had the highest rate of harm (9.16%), while Francophones admitted to these same hospitals had the lowest rate of harm (5.93%). In the unadjusted analysis, Francophones were less likely to experience harm in bilingual hospitals than in hospitals that were not required by law to provide services in French (*English-speaking hospitals)* (RR = 0.88, *p* = 0.048); the opposite was true for Anglophones and Allophones, who were more likely to experience harm in bilingual hospitals (RR = 1.17, *p* <  0.001 and RR = 1.41, *p* <  0.001, respectively). The risk of harm was not significant in the adjusted analysis.

**Conclusions:**

Home care recipients residing in Eastern and North-Eastern Ontario were more likely to experience harm in language-discordant hospitals, but the risk of harm did not persist after adjusting for confounding variables.

## Background

Linguistic diversity in North America is increasing at an unprecedented rate. In the United States, approximately 67 million people (22% of the population) primarily speak a language other than English, of which 39% speak English less than “very well” [1]. The situation is similar in Canada, where almost 9 million people (25% of the population) live in a province that does not recognize their mother tongue as an official language [2]. Despite government-led initiatives to improve the delivery of healthcare services to minority linguistic groups, health disparities related to access, quality, and safety of care persist across linguistic groups [3–6].

There is a growing body of evidence that language barriers may contribute to the health disparities observed across linguistic groups. The majority of previous studies have considered the relationship between a patient’s linguistic group and specific health-related outcomes. For instance, studies have shown that linguistic groups who live in minority language situations face barriers when accessing healthcare services [7, 8], have longer emergency department visits and hospitals stays [9, 10], receive lower quality discharge documentation and discharge instructions [11, 12], have higher rates of hospital admissions and re-admissions [13, 14], and experience more harmful events in hospitals [15–18]. However, due to limitations regarding inaccurate measurement of patients’ primary language and inadequate adjustment of confounding variables, it is not known whether the differences in these studies are due to language barriers, or to systematic differences in patient characteristics (e.g., socioeconomic status) and/or health status across linguistic groups.

Few studies have directly assessed the impact of patient-provider language discordance, which occurs when the patient and their provider lack proficiency in a shared language. Studies conducted in the United States show that Spanish-speaking patients are more likely to attend scheduled office appointments [19], ask questions during appointments [20, 21], report better health outcomes [22], receive more health education [23–25], and have better recall of health information [20, 23] when cared for by Spanish-speaking physicians. Language-concordant care has been shown to result in objective benefits for specific health outcomes related to chronic disease management, such as better control of diastolic blood pressure [26], glycemia [26–28], and low-density-lipoprotein levels [26], among patients with type 2 diabetes. Another study found an increase in 1-year all-cause mortality among Canadian immigrants treated for tuberculosis in the presence of patient-provider language discordance, when compared to immigrants treated by physicians who were proficient in their language [29].

To our knowledge, no population-level study has considered the relationship between patient-provider language discordance and the risk of harmful events in acute care settings. In Canada, one out of 18 patients admitted to hospital experiences a harmful event, which is generally defined as an unintended outcome with negative consequences that could potentially be prevented with evidence-based practices [30]. The purpose of this study was to compare the incidence of in-hospital harm across linguistic groups in Ontario, Canada, and determine whether admission to a language-discordant hospital is a risk factor for in-hospital harm, before and after adjusting for potentially confounding variables.

## Methods

### Study design and population

We conducted a population-based retrospective cohort study in Ontario, Canada, using administrative databases. We included Ontario residents who received publicly funded home care services from April 1, 2007 to March 31, 2015 and who were hospitalized within 2 years of their first home care assessment, conducted using the Resident Assessment Instrument for Home Care (RAI-HC) [31]. Respondents of the RAI-HC are typically frail (e.g., have limitations in activities in daily living and limited life expectancy), and are assessed for language status [32, 33]. We excluded residents who were older than 105 years of age at the time of the study or not eligible for Ontario’s universal health insurance plan (i.e., the Ontario Health Insurance Plan (OHIP)) during the study period. In 2007, the planning and distribution of public healthcare services in Ontario was divided into 14 geographically defined local health integration networks (LHINs) [34]. We included home care recipients who resided in the Champlain and North-East LHINs; this allowed us to consider regions where Francophones (according to mother tongue) represent an important proportion of the population (18.5% in the Champlain LHIN and 22.3% in the North-East LHIN according to the 2016 Canadian Census). By comparison, the proportion of Francophones in all other LHINs is less than 3.1%, and Allophones (i.e., residents whose mother tongue is not English or French) represent 16.8 and 6.2% of the population in the Champlain and North-East LHINs, respectively [2].

The RAI-HC database, which captures the baseline characteristics of Ontario residents receiving publicly funded home care services for at least 60 consecutive days or while waiting for admission into long-term care [35], allowed us to study a cohort of frail, older residents with an increased risk of hospitalization and harm [32, 33]. Residents who completed more than one home care assessment were indexed at the time of their first assessment and followed for 2 years. Thus, all home care recipients appeared once in the baseline cohort and were censored at 2 years or at death (whichever occurred first). Patients with multiple hospitalizations were considered as separate entries when calculating the incidence of harm (i.e., one entry per hospitalization) in order to capture the total incidence of harm during the follow-up period.

### Data sources

Baseline characteristics were obtained from the following databases: RAI-HC database; Registered Persons Database, which captures resident-level information, such as age, sex, and postal code; and Facilities Database, which captures hospital-level information for all institutions funded by the Ministry of Health and Long-Term Care. Patient and hospital postal codes were linked to the 2011 Statistics Canada Census to obtain income quintile and urban/rural status at the level of the patients and hospitals, respectively [36]. Chronic conditions were identified using algorithms validated by ICES (formerly known as the Institute for Clinical Evaluative Sciences) and applied in previous studies (see Additional file 1) [33, 37–39]. Outcomes were obtained from the Discharge Abstract Database (DAD), which captures information on all admissions to acute care hospitals.

### Exposure

Patient language was obtained from the RAI-HC home care assessment, where interviewers record the language that the home care recipient primarily speaks and/or understands. Interviewers also consult clinical records, family members, and referral sources to identify home care recipients who need an interpreter [31]. We considered three main linguistic groups: Anglophone (i.e., primary language English), Francophone (i.e., primary language French), and Allophone (i.e., primary language is a language other than English or French).

Hospital language was defined using language designation status according to the *French Language Services Act* [40], which is a provincial law that requires government agencies to provide all of their services in both French and English. Currently, 12 acute care hospitals in Ontario (4 in Champlain LHIN and 8 in North-East LHIN) are required by law to provide all of their services in both French and English. The remaining 147 acute care hospitals are only required by law to provide all of their services in English. We defined these hospitals as “bilingual hospitals” and “English-speaking hospitals,” respectively.

### Outcomes

In-hospital harmful events were identified using the Hospital Harm Indicator developed by the Canadian Institute for Health Information [30]. The Hospital Harm Indicator uses *International Statistical Classification of Diseases and Related Health Problems, 10th Revision, Canada* codes recorded in the DAD to identify harmful events occurring during a hospitalization (Additional file 2). The indicator combines information from diagnosis clusters, diagnosis codes, diagnosis types, and intervention codes to classify each in-hospital harmful event into one of the following four categories: i) harm from general medical care, including medication administration (e.g., electrolyte and fluid imbalance, delirium); ii) infections (e.g., urinary tract infections, pneumonia); iii) patient accidents (e.g., falls); and, iv) harm from procedures (e.g., anemia or hemorrhage due to medical or surgical care, laceration/puncture) [30]. With the exception of patient accidents, every category of harmful events can be divided into additional subcategories, for a total of 31 subcategories, which are provided in Additional file 2 [30]. We defined a “harmful hospitalization” as a hospitalization with at least one harmful event. Some patients experienced multiple harmful events during a single hospitalization. In these cases, we assigned each harmful event to its respective category, and we treated the hospitalization as a single harmful hospitalization. Thus, a harmful hospitalization could include harm from health care-associated conditions, infections, patient accidents, and/or procedures.

### Statistical analysis

We performed descriptive analyses to compare patient and hospital characteristics across linguistic groups and hospital language, respectively. Means (+/− standard deviation) were reported for continuous variables, and proportions were reported for categorical variables. Baseline characteristics were compared using analysis of variance (ANOVA) for continuous variables and chi-squared tests for categorical variables.

We compared outcomes across hospitals (i.e., English-speaking and bilingual) after stratifying by patient language. We considered three linguistic groups (i.e., Anglophone, Francophone, Allophone) in three separate models to determine the impact of hospital language on the rate of harm within each linguistic group. Thus, each model compared outcomes for patients admitted to bilingual hospitals to those admitted to English-speaking hospitals for a given linguistic group. Patients in English-speaking hospitals were the reference group in all three models.

We obtained both unadjusted and adjusted estimates of the relative risk. Unadjusted estimates were calculated using the method described by Katz et al. [41], while adjusted estimates were obtained using a modified Poisson regression with generalized estimating equations to account for hospital-level clustering [42–44]. Adjusted regression analyses included potential confounders related to both patient characteristics (age at admission, sex, marital status, education, neighbourhood-level income quintile, urban/rural residence, Charlson Comorbidity Index [45], Activities of Daily Living (ADL) scale [46], Cognitive Performance Scale [47]) and hospital characteristics (academic institution (yes/no), emergency department at hospital (yes/no), urban/rural status of hospital, number of ICU beds, number of medical beds, number of obstetric beds, number of pediatric beds, and number of surgical beds). We excluded observations with incomplete or missing data for primary language (*n* = 3). Statistical tests were two-tailed, and the significance threshold was set at 0.05.

### Ethics approval

ICES (formerly known as the Institute for Clinical Evaluative Sciences) is a prescribed entity under section 45 of Ontario’s *Personal Health Information Protection Act*. Section 45 authorizes ICES to collect personal health information, without consent, for the purpose of analysis or compiling statistical information with respect to the management of, evaluation or monitoring of, allocation of resources to or planning for all or part of the health system. This project was conducted under section 45 and approved by the ICES Privacy and Compliance Office.

## Results

### Baseline characteristics

We included 50,267 long-stay home care recipients residing in the Champlain and North-East LHINs who met eligibility criteria. The majority of home care recipients were Anglophone (77.5%), while Francophones and Allophones represented 16.0 and 6.4% of the cohort, respectively. The baseline characteristics of Anglophones, Francophones, and Allophones are presented in Table 1. Compared to Anglophones and Francophones, Allophones tended to be older and were more likely to be married or in a common-law relationship. There was a greater proportion of females among Francophones than among Anglophones and Allophones. Both Francophones and Allophones were less likely to have completed high school than Anglophones. The proportion of residents living in lower-income neighbourhoods and in rural areas was greater for Francophones than both Anglophones and Allophones. Anglophones and Allophones had a similar distribution of neighbourhood income levels, while a greater proportion of Allophones lived in urban areas.

**Table 1:** Baseline characteristics of hospitalized home care recipients, by patient linguistic group

Baseline Characteristic	Anglophone (*N* = 38,975)	Francophone (*N* = 8053)	Allophone (*N* = 3239)	***P*** value*
**Age at admission (years) – mean +/− s.d.**	76.6 ± 13.1	77.1 ± 11.9	80.6 ± 9.2	< 0.001
**Sex – no. (%)**				
Female – no. (%)	22,458 (57.6%)	4765 (59.2%)	1842 (56.9%)	0.002
Male – no. (%)	16,517 (42.4%)	3288 (40.8%)	1397 (43.1%)	
**Marital Status – no. (%)**				
Not Married	22,812 (58.5%)	4801 (59.6%)	1770 (54.6%)	< 0.001
Married or Common-Law	15,503 (39.8%)	3133 (38.9%)	1446 (44.6%)	
Other	660 (1.7%)	119 (1.5%)	23 (0.7%)	
**Education – no. (%)**				
Less than High School	13,179 (33.8%)	4268 (53.0%)	1419 (43.8%)	< 0.001
High School	6566 (16.8%)	833 (10.3%)	251 (7.7%)	
Some Post-Secondary	5598 (14.4%)	697 (8.7%)	242 (7.5%)	
University Graduate	3994 (10.2%)	425 (5.3%)	212 (6.5%)	
Missing	9638 (24.7%)	1830 (22.7%)	1115 (34.4%)	
**Income Quintile – no. (%)**				
1 (lowest)	10,054 (25.8%)	2313 (28.7%)	883 (27.3%)	< 0.001
2	7981 (20.5%)	1857 (23.1%)	672 (20.7%)	
3	7446 (19.1%)	1615 (20.1%)	580 (17.9%)	
4	7328 (18.8%)	1303 (16.2%)	576 (17.8%)	
5 (highest)	6051 (15.5%)	944 (11.7%)	513 (15.8%)	
Missing	115 (0.3%)	21 (0.3%)	15 (0.5%)	
**Urban/Rural Residence – no. (%)**				
Urban	28,089 (72.1%)	5420 (67.3%)	2867 (88.5%)	< 0.001
Rural	10,868 (27.9%)	2630 (32.7%)	372 (11.5%)	
Missing	18 (0.0%)	< 5 (0.0%)	N/A	

* Baseline characteristics were compared using analysis of variance (ANOVA) for continuous variables and chi-squared tests for categorical variables

The functional status and health characteristics of the cohort are presented in Table 2. Francophones tended to have more chronic conditions when compared to Anglophones and Allophones (*p* = 0.010). However, there was no significant difference in measures of comorbidity (Charlson Comorbidity Index; *p* = 0.319) or health decline (Changes in Health, End-Stage Disease, Signs and Symptoms (CHESS) score; *p* = 0.213) across linguistic groups. Anglophones and Francophones were similar in terms of functional status and cognitive performance, while Allophones were less likely to perform their activities of daily living independently (*p* <  0.001) and more likely to have cognitive impairment (*p* <  0.001).

**Table 2:** Functional status & health characteristics of hospitalized home care, by patient linguistic group

Functional Status & Health Characteristic	Anglophone (*N* = 38,975)	Francophone (*N* = 8053)	Allophone (*N* = 3239)	***P*** value*
**Number of Chronic Conditions – mean +/− s.d.**	4.26 ± 2.04	4.33 ± 2.09	4.22 ± 1.98	0.010
**Charlson Comorbidity Index – mean +/− s.d.**	2.07 ± 2.15	2.07 ± 2.09	2.13 ± 2.08	0.319
**Activities of Daily Living Scale – no. (%)**				
Independent	24,356 (62.5%)	4986 (61.9%)	1637 (50.5%)	< 0.001
Supervision Required	4142 (10.6%)	869 (10.8%)	415 (12.8%)	
Limited Impairment	5546 (14.2%)	1184 (14.7%)	588 (18.2%)	
Extensive Assistance Required (1)	2422 (6.2%)	529 (6.6%)	287 (8.9%)	
Extensive Assistance Required (2)	1450 (3.7%)	283 (3.5%)	180 (5.6%)	
Dependent	885 (2.3%)	173 (2.1%)	101 (3.1%)	
Total Dependence	174 (0.4%)	29 (0.4%)	31 (1.0%)	
**Cognitive Performance Scale – no. (%)**				
Intact	15,555 (39.9%)	3087 (38.3%)	984 (30.4%)	< 0.001
Borderline Intact	6634 (17.0%)	1413 (17.5%)	558 (17.2%)	
Mild Impairment	6830 (17.5%)	1513 (18.8%)	615 (19.0%)	
Moderate Impairment	8218 (21.1%)	1626 (20.2%)	808 (24.9%)	
Moderate Severe Impairment	577 (1.5%)	109 (1.4%)	94 (2.9%)	
Severe Impairment	1029 (2.6%)	279 (3.5%)	149 (4.6%)	
Very Severe Impairment	132 (0.3%)	26 (0.3%)	31 (1.0%)	
**CHESS Score – no. (%)**				
No Health Instability	8095 (20.8%)	1589 (19.7%)	689 (21.3%)	0.213
Minimal Health Instability	11,570 (29.7%)	2370 (29.4%)	977 (30.2%)	
Low Health Instability	10,969 (28.1%)	2302 (28.6%)	919 (28.4%)	
Moderate Health Instability	6575 (16.9%)	1403 (17.4%)	496 (15.3%)	
High Health Instability	1681 (4.3%)	369 (4.6%)	151 (4.7%)	
Very High Health Instability	85 (0.2%)	20 (0.2%)	7 (0.2%)	

Note: *CHESS* Changes in Health, End-Stage Disease, Signs, and Symptoms, *COPD* Chronic Obstructive Pulmonary Disease

* Functional status & health characteristics were compared using analysis of variance (ANOVA) for continuous variables and chi-squared tests for categorical variables

We identified 159 acute care hospitals in the Champlain and North-East LHINs. Of these hospitals, 12 (7.5%) were required by law to provide all of their services in both French and English (*bilingual hospitals*), while 147 (92.5%) were only required by law to provide all of their services in English (*English-speaking hospitals*). The characteristics of the hospitals are presented in Table 3. Bilingual hospitals were more likely to be academic institutions, to have an emergency department, and to be located in rural areas when compared to English-speaking hospitals. Bilingual hospitals also tended to have fewer beds overall, and within each unit (intensive care unit, medicine, obstetrics, pediatrics, surgery). However, none of these differences were significant at the 0.05 level.

**Table 3:** Characteristics of hospitals, by hospital language

Characteristic of Hospital	Language of Hospital		***P*** value*
	English-speaking (*N* = 147)	Bilingual (*N* = 12)	
**Academic Institution – no. (%)**			
Yes	19 (12.9%)	3 (25.0%)	0.244
No	128 (87.1%)	9 (75.0%)	
**Emergency Department – no. (%)**			
Yes	140 (95.2%)	12 (100%)	0.439
No	7 (4.8%)	0 (0%)	
**Urban/Rural Hospital – no. (%**			
Urban	74 (50.3%)	4 (33.3%)	0.257
Rural	73 (49.7%)	8 (66.7%)	
**Total Number of Beds – mean +/− s.d.**	121.85 ± 129.42	47.44 ± 48.57	0.089
Number of ICU beds – mean +/− s.d.	11.20 ± 15.76	2.44 ± 3.32	0.100
Number of medical beds – mean +/− s.d.	51.65 ± 65.69	17.56 ± 26.42	0.124
Number of obstetric beds – mean +/− s.d.	9.78 ± 13.47	6.11 ± 9.24	0.424
Number of pediatric beds – mean +/− s.d.	4.21 ± 7.24	1.33 ± 1.80	0.238
Number of surgical beds – mean +/− s.d.	28.70 ± 42.42	9.67 ± 16.29	0.184

Note: *ICU* Intensive Care Unit

* Characteristics were compared using t-tests for continuous variables and chi-squared tests for categorical variables

### Harmful hospitalizations

We identified 106,879 hospitalizations during the 2-year follow-up period. A total of 6788 (6.35%) hospitalizations included at least one harmful event (Table 4). The proportion of harmful hospitalizations experienced by Allophones (7.63%) was greater than that experienced by Anglophones (6.29%; *p* <  0.001) and Francophones (6.15%; *p* <  0.001). The difference in the rate of harmful hospitalizations when comparing Anglophones and Francophones was not significant (*p* = 0.492). Francophone hospitals had a higher proportion of harmful hospitalizations than anglophone hospitals (6.80% vs. 6.05%; *p* <  0.001).

**Table 4:** Rate of harmful hospitalization, by patient linguistic group and hospital language*

Hospital Language	Patient Linguistic Group			***P*** value
	Anglophone (*N* = 83,515)	Francophone (*N* = 16,873)	Allophone (*N* = 6491)	
**Overall Harmful Hospitalizations**	5255 (6.29%)	1038 (6.15%)	495 (7.63%)	< 0.001
**Harmful Hospitalizations by Hospital Language**				
English-speaking (N = 147)	3318 (5.96%)	309 (6.75%)	243 (6.50%)	0.048
Bilingual (N = 12)	1937 (6.96%)	729 (5.93%)	252 (9.16%)	< 0.001
**P value†**	< 0.001	0.048	< 0.001	

* Bold values denote significance at the 0.05 level

† Proportions of harmful hospitalizations were compared across patient linguistic groups and hospital language using chi-squared tests

Overall, Allophones admitted to bilingual hospitals had the highest rate of harm (9.16%), while Francophones admitted to these same hospitals had the lowest rate of harm (5.93%), a 1.5-fold relative difference. In English-speaking hospitals, the proportion of harmful hospitalizations was lowest for Anglophones (5.96%) and highest for Francophones (6.75%), while Allophones experienced an intermediate rate of harm (6.50%).

### Risk of harm by patient and hospital language

**Fig. 1:**
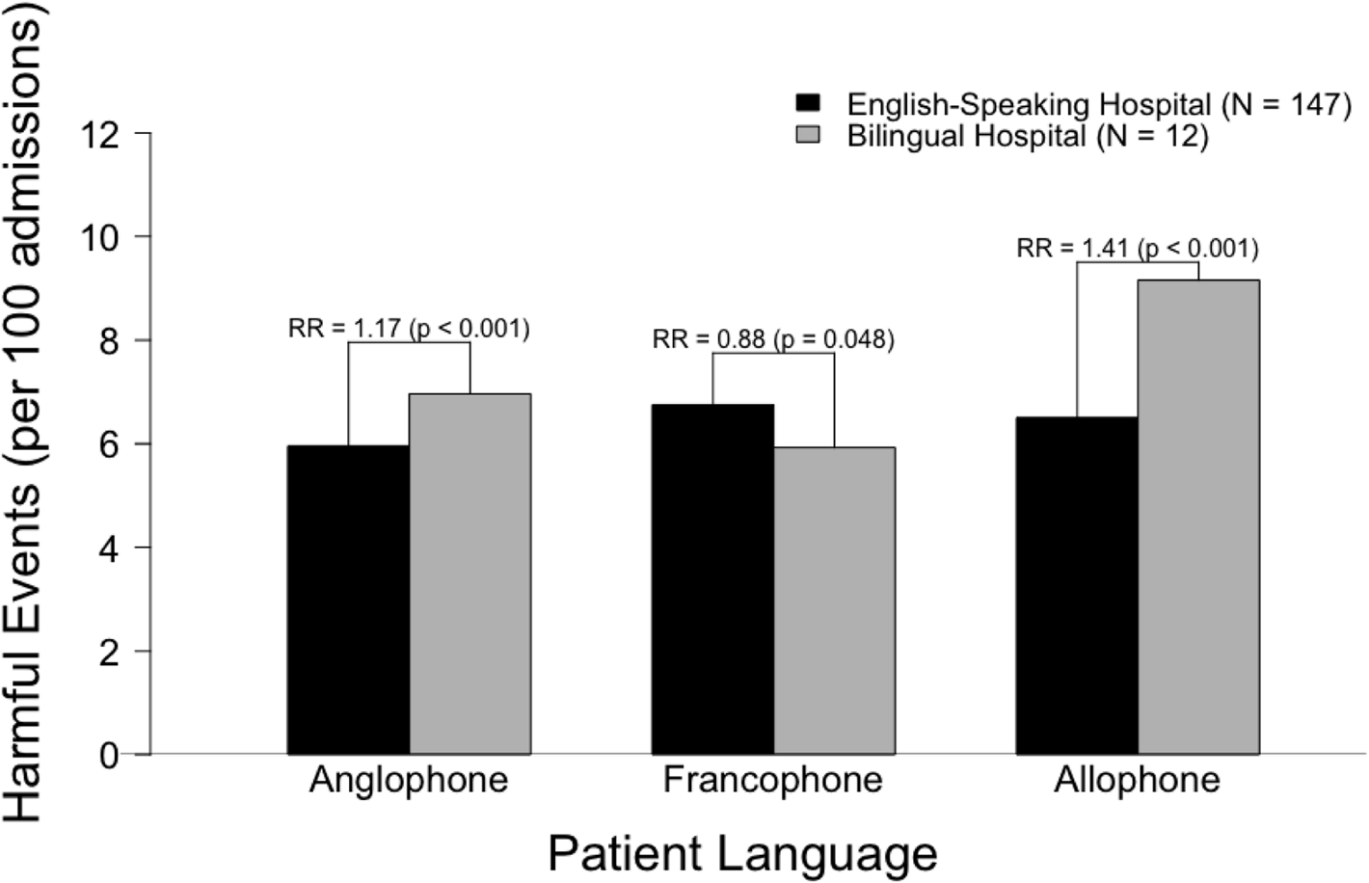
Unadjusted relative risk of harm, stratified by hospital language

**Fig. 2:**
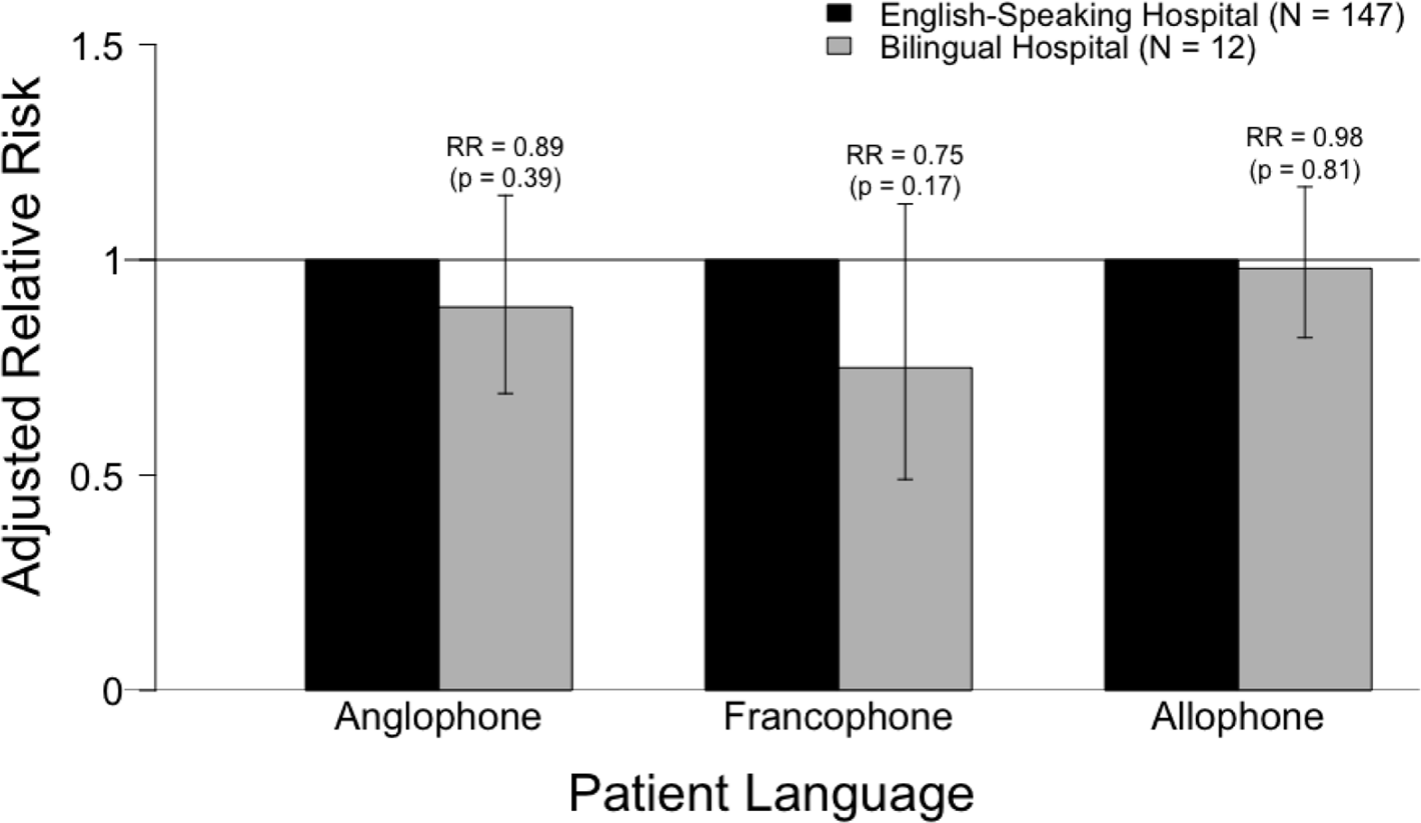
Adjusted relative risk of harm, stratified by hospital language*. * Adjusted for age at admission, sex, marital status, education, neighbourhood-level income quintile, urban/rural residence, Charlson Comorbidity Index, Activities of Daily Living (ADL) scale, Cognitive Performance Scale, academic institution (yes/no), emergency department at hospital (yes/no), urban/rural hospital, number of ICU beds, number of medical beds, number of obstetric beds, number of pediatric beds, number of surgical beds

In the unadjusted analysis, Anglophones and Allophones were more likely to experience harm in bilingual hospitals than in English-speaking hospitals (RR = 1.17, 95% CI 1.11–1.23 and RR = 1.41, 95% CI 1.19–1.67, respectively), while Francophones were less likely to experience harm in bilingual hospitals (RR = 0.88, 95% CI 0.77–0.99) (Fig. 1). After adjusting for potential confounders, the relative risk of harm was not significant when comparing patients admitted to bilingual and English-speaking hospitals within each linguistic group (Table 5); Anglophones and Allophones were equally likely to experience harm in bilingual hospitals as they were in English-speaking hospitals (RR = 0.89, 95% CI 0.69–1.15 and RR = 0.98, 95% CI 0.82–1.17, respectively). For Francophones, the protective effect of being treated in a bilingual hospital did not persist in the adjusted analysis, but the point estimate was further from 1 than in the unadjusted analysis (RR = 0.88, 95% CI 0.77–0.99, adjusted RR = 0.75, 95% CI 0.49–1.13) (Fig. 2).

**Table 5:** Relative risk of experiencing a harmful hospitalization, stratified by hospital language*

	Model 1: Anglophones		Model 2: Francophones		Model 3: Allophones	
	English-speaking Hospital	Bilingual Hospital	English-speaking Hospital	Bilingual Hospital	English-speaking Hospital	Bilingual Hospital
Unadjusted Relative Risk (95% CI)	1.0 (ref)	**1.17 (1.11–1.23)**	1.0 (ref)	**0.88 (0.77–0.99)**	1.0 (ref)	**1.41 (1.19–1.67)**
Adjusted Relative Risk (95% CI)†	1.0 (ref)	0.89 (0.69–1.15)	1.0 (ref)	0.75 (0.49–1.13)	1.0 (ref)	0.98 (0.82–1.17)

* Bold values denote significance at the 0.05 level

† Adjusted for age at admission, sex, marital status, education, neighbourhood-level income quintile, urban/rural residence, Charlson Comorbidity Index, Activities of Daily Living (ADL) scale, Cognitive Performance Scale, academic institution (yes/no), emergency department at hospital (yes/no), urban/rural hospital, number of ICU beds, number of medical beds, number of obstetric beds, number of pediatric beds, number of surgical beds

## Discussion

In this study, Allophones (7.63%) were more likely to experience harm than Anglophones (6.29%) and Francophones (6.15%). The rate of harm was influenced by both patient language and hospital language. Allophones admitted to hospitals that were required by law to provide services in both French and English (i.e., *bilingual hospitals*) had the highest rate of harm (9.16%), which represents a 54% increase in relative risk when compared to Anglophones admitted to hospitals that were not required to provide services in French (i.e., *English-speaking hospitals*). Francophones were 12% less likely to experience harm in bilingual hospitals than in English-speaking hospitals, while Anglophones and Allophones were 17 and 41% more likely to experience harm in bilingual hospitals. After adjusting for potential confounders, which included both patient and hospital characteristics, there was no difference in the risk of harm when comparing patients from any linguistic group across hospitals stratified by language.

In Ontario, a total of 12 acute care hospitals are required by the *French Language Services Act* to provide all of their services in English and French [40]; we defined these hospitals as “bilingual hospitals” [40]. All hospitals may provide services in languages other than English (including French), but bilingual hospitals are the only hospitals that are required by law to do so. Francophones in these hospitals must be represented at all levels of management (e.g., human resources, senior management, and board of directors), and health care providers must actively offer services in French to their patients [48]. We hypothesized that Francophones would experience less harm in these hospitals, in part, because of improved patient-provider communication. As a result of the requirements imposed on bilingual hospitals, a larger proportion of health care providers in these hospitals are able to communicate in French (when compared to health care providers in other hospitals). Thus, fewer Francophone patients should face language barriers in bilingual hospitals. Previous studies have shown that patients who are treated in language-discordant environments are more likely to have poor health outcomes [26–29], although the impact of patient-provider language-discordance on the risk of harm has never been considered directly. Thus, it is possible that the lower risk of harm can be explained, to a certain extent, by the protective effects of being treated in a language-concordant environment. However, since the risk of harm did not persist after adjusting for confounding variables, differences in the rates of harm cannot be attributed to linguistic factors alone.

We hypothesized that health care providers in bilingual hospitals would have a better understanding of the needs of linguistic minorities, given that Francophones are a minority linguistic group in Ontario. Thus, we believed that increased awareness and knowledge regarding the negative impacts of language barriers among health care providers in bilingual hospitals would translate to better outcomes for Allophones. However, Allophones were more likely to experience harm in bilingual hospitals than in English-speaking hospitals in the unadjusted analysis, and equally likely to experience harm in both bilingual and English-speaking hospitals in the adjusted analysis. According to the 2016 Canadian Census, 87.4% of Ontarians whose mother tongue is neither English nor French can conduct a conversation in English, while only 15.6% can conduct a conversation in French [2]. Thus, a larger proportion of Allophones can speak English than French, which means that Allophones may experience more communication problems in bilingual hospitals. This could explain why Allophones did not experience more harm in English-speaking hospitals.

The risk of harm across bilingual and English-speaking hospitals did not persist in the adjusted analysis for any of the linguistic groups. It is possible that Anglophones and Allophones admitted to bilingual hospitals had a greater risk of experiencing harm (in the unadjusted analysis) because of systematic differences compared to patients of the same linguistic group admitted to English-speaking hospitals. Similarly, Francophones admitted to English-speaking hospitals may have had more risk factors for harm at baseline. Bilingual hospitals also had a higher rate of harm overall than English-speaking hospitals. This could be due to the fact that a greater proportion of bilingual hospitals are academic institutions, which generally have a higher rate of harm [49, 50]. While this could provide an explanation for the increased risk of harm for Anglophones and Allophones in bilingual hospitals, it does not explain the decreased risk observed for Francophones admitted to bilingual hospitals in the unadjusted analysis. Furthermore, while the adjusted relative risk of harm for Francophones in bilingual hospitals compared to English-speaking hospitals was not significant, the point estimate was further from 1 than the unadjusted estimate, suggesting a protective effect.

### Limitations

We obtained primary language from RAI-HC home care assessments. During these assessments, interviewers determine the home care recipient’s primary language by listening and observing and, if necessary, by asking the home care recipient to specify his or her primary language [31]. Many Ontarians speak multiple languages, and it is unclear how interviewers assigned primary language for such patients. Furthermore, the interviewers do not assess language proficiency, which is particularly important for multilingual patients. However, recent analyses performed by our group have shown that the language variable in the RAI-HC Database has a high level of agreement (kappa = 0.76) with the language most often spoken at home, as determined by the Canadian Community Health Survey (Batista et al., unpublished data, 2019). Finally, since language is recorded prior to admission to hospital, misclassification of the exposure should be non-differential, leading the results to be biased towards the null.

Our interpretation of the results assumed that health care providers in bilingual and English-speaking hospitals had a higher level of proficiency in French and English, respectively, when compared to health care providers in hospitals with the opposite language status. Physicians who practice in Ontario can speak more than 130 different languages [51], and many physicians can speak multiple languages. Thus, many patients, especially Anglophones and Francophones, are likely to receive health care services in their primary language even in hospitals that are not required by law to do so. However, since the language employed during patient-provider encounters is not recorded, we do not know whether individual encounters occurred in the patient’s primary language, and we cannot account for this in our analysis. Some hospitals are required by law to provide some (but not all) of their services in both French and English (*partially bilingual hospitals*) [40]. In this study, we compared rates of harm in hospitals required by law to provide all of their services in English and French to other hospitals, including partially bilingual hospitals. As a result, any effect attributed to patient-provider language barriers is likely underestimated, since some of the hospitals identified in this study as English-speaking (4 in Champlain LHIN and 6 in North-East LHIN) are in fact required by law to provide certain services in both French and English. Finally, our study was conducted in two geographic regions within a Canadian province with one official language (English) and one language for which the provision of services is guaranteed within certain hospitals (French). To our knowledge, no other jurisdiction in North America guarantees the provision of health care services to minority linguistic groups to the extent that the *French Language Services Act* guarantees access to services in French. Thus, our results may not be generalizable to other regions within Ontario or Canada or to populations with different legislation regarding language rights (e.g., Anglophone minority in Quebec and Francophone minority outside of Ontario).

## Conclusions

In this study, Allophone home care recipients residing in Eastern and North-Eastern Ontario were more likely to experience a harmful hospitalization than Anglophones and Francophones. Furthermore, patients were less likely to experience harm in language-concordant hospitals (i.e., Anglophones and Allophones in English-speaking hospitals and Francophones in bilingual hospitals). However, the risk of harm due to language-discordance did not persist after adjusting for confounding variables. Thus, the differences in the rates of harm cannot be attributed to linguistic factors alone; the disparities are likely due, at least in part, to differences in patient and/or hospital characteristics. Further research is needed to understand the mechanism that gives rise to these disparities.

## Supplementary information

**Additional file 1.** Chronic conditions. Description of chronic conditions identified using algorithms validated by ICES.

**Additional file 2.** Categories and subcategories of harmful events. Description of in-hospital harmful events identified using the Hospital Harm Indicator.

## Data Availability

The data that support the findings of this study are available from ICES but restrictions apply to the availability of these data, which were used under license for the current study, and so are not publicly available. Data are however available from the authors upon reasonable request and with permission of ICES.

## Abbreviations

ADL: Activities of Daily Living
ANOVA: Analysis of Variance
CHESS: Changes in Health, End-Stage Disease, Signs and Symptoms
DAD: Discharge Abstract Database
ICES: Institute for Clinical Evaluative Sciences
OHIP: Ontario Health Insurance Plan
RAI-HC: Resident Assessment Instrument for Home Care
