# Supplementary material for: The impact of hospital language on the rate of in-hospital harm. A retrospective cohort study of home care recipients in Ontario, Canada
Source: BMC Health Serv Res. 2020 Apr 21;20:340. doi: 10.1186/s12913-020-05213-6 (PMC7175496; doi:10.1186/s12913-020-05213-6)
Supplement: Supplementary file 1 — Additional file 1. Chronic conditions. Description of chronic conditions identified using algorithms validated by ICES. [file 12913_2020_5213_MOESM1_ESM.docx]

**Appendix 1. Chronic conditions**

We identified a total of 18 chronic conditions using administrative data from April 1, 2001 onwards.

We used validated algorithms to ascertain cases of the following 8 chronic conditions:

1. Acute myocardial infarction
2. Asthma
3. Congestive Heart Failure
4. COPD
5. Dementia
6. Diabetes
7. Hypertension
8. Rheumatoid Arthritis

The remaining 10 chronic conditions were defined according to inpatient hospital diagnostic codes (at least 1 from Discharge Abstract Database) or outpatient physician billing codes (at least 2 from Ontario Health Insurance Plan within a 2-year period):

1. Arrhythmia
2. Cancer
3. Coronary Heart Disease
4. IBD
5. Non-psychotic mood and anxiety disorders
6. Osteoarthritis
7. Osteoporosis
8. Other mental health conditions
9. Renal Disease
10. Stroke
