# Supplementary material for: The impact of hospital language on the rate of in-hospital harm. A retrospective cohort study of home care recipients in Ontario, Canada
Source: BMC Health Serv Res. 2020 Apr 21;20:340. doi: 10.1186/s12913-020-05213-6 (PMC7175496; doi:10.1186/s12913-020-05213-6)
Supplement: Supplementary file 2 — Additional file 2. Categories and subcategories of harmful events. Description of in-hospital harmful events identified using the Hospital Harm Indicator. [file 12913_2020_5213_MOESM2_ESM.docx]

**Appendix 2. Categories and subcategories of harmful events**

In-hospital harmful events were identified using the Hospital Harm Indicator developed by the Canadian Institute for Health Information (1). The Hospital Harm Indicator uses *International Statistical Classification of Diseases and Related Health Problems, 10^th^ Revision, Canada* codes recorded in the Discharge Abstract Database to identify harmful events occurring during a hospitalization. The indicator combines information from diagnosis clusters, diagnosis codes, diagnosis types, and intervention codes to classify each in-hospital harmful event into one of the following four categories: harm from general medical care (e.g., electrolyte and fluid imbalance, delirium), infections (e.g., urinary tract infections, pneumonia), patient accidents (e.g., falls, trauma), harm from procedures (e.g., anemia or hemorrhage, laceration and/or puncture). The categories of harmful events can be divided into additional subcategories, for a total of 31 subcategories.

Harm from general medical care (11 subcategories): harm related to health care provided or medications administered

1. **Anemia or hemorrhage**, excluding anemia or hemorrhage due to medical or surgical care
2. **Obstetric hemorrhage** related to non-instrument assisted vaginal delivery
3. **Obstetric trauma** (i.e., injuries to the mother) related to non-instrument assisted vaginal delivery
4. **Birth trauma** (i.e., injuries to the newborn) related to non-instrument assisted vaginal delivery
5. Delirium
6. **Venous thromboembolism**: embolism, phlebitis, thrombosis.
7. **Altered blood glucose levels with complications**: hypoglycemia, lactic acidosis.
8. **Pressure ulcer**
9. **Electrolyte and fluid imbalance**, including acid-base imbalance.
10. **Medication incidents:** incorrect administration of medications
11. **Infusion, transfusion, and injection complications**

Patient accidents (1 subcategory): injuries that occur during a hospital stay that are not directly related to health care provided or to a surgical procedure

1. Patient trauma

Infections (7 subcategories): infections that occur during a hospital stay

1. **Urinary tract infections**
2. **Post-procedural infections:** all infections related to a medical or surgical procedure.
3. **Gastroenteritis**, excluding gastrointestinal infections those due to *Clostritium difficile*
4. **Pneumonia**, excluding aspiration pneumonia
5. **Aspiration pneumonia**
6. **Sepsis**, excluding neonatal sepsis
7. Infections due to *Clostridium difficile*, MRSA, VRE

Harm from procedures (12 subcategories): abnormal reactions, complications, and misadventures that occur during a medical or surgical procedure

1. **Anemia or hemorrhage** due to medical or surgical care
2. **Obstetric hemorrhage** related to instrument assisted vaginal delivery or Caesarian section delivery
3. **Obstetric trauma** (i.e., injuries to the mother) related to instrument assisted vaginal delivery or Caesarian section delivery
4. **Birth trauma** (i.e., injuries to the newborn) related to instrument assisted vaginal delivery or Caesarian section delivery
5. **Patient trauma** related to a medical or surgical procedure
6. **Device failure**: mechanical complications of medical devices related to a medical or surgical procedure.
7. **Laceration/puncture**: accidental or unintended laceration/puncture related to a medical or surgical procedure
8. **Pneumothorax** related to a medical or surgical procedure
9. **Wound Disruption**: disruption of obstetric wound or surgical wound
10. **Retained foreign body**: foreign body or substance unintentionally left in the body during a medical or surgical procedure
11. **Post-procedural shock**
12. **Selected serious events**: harm related serious patient safety events, such as failure in ligature or suture, failure of sterile precautions, performance of inappropriate operation, wrong placement of endotracheal tube.

Reference: Canadian Institute for Health Information. Measuring Patient Harm in Canadian Hospitals: Technical Report. Ottawa, Canada; 2016.
